# TRP Channels as Potential Targets for Sex-Related Differences in Migraine Pain

**DOI:** 10.3389/fmolb.2018.00073

**Published:** 2018-08-14

**Authors:** Maite Artero-Morales, Sara González-Rodríguez, Antonio Ferrer-Montiel

**Affiliations:** Instituto de Biología Molecular y Celular, Universitas Miguel Hernández, Elche, Spain

**Keywords:** TRP channels, TRPV1, sex hormones, estrogens, migraine

## Abstract

Chronic pain is one of the most debilitating human diseases and represents a social and economic burden for our society. Great efforts are being made to understand the molecular and cellular mechanisms underlying the pathophysiology of pain transduction. It is particularly noteworthy that some types of chronic pain, such as migraine, display a remarkable sex dimorphism, being up to three times more prevalent in women than in men. This gender prevalence in migraine appears to be related to sex differences arising from both gonadal and genetic factors. Indeed, the functionality of the somatosensory, immune, and endothelial systems seems modulated by sex hormones, as well as by X-linked genes differentially expressed during development. Here, we review the current data on the modulation of the somatosensory system functionality by gonadal hormones. Although this is still an area that requires intense investigation, there is evidence suggesting a direct regulation of nociceptor activity by sex hormones at the transcriptional, translational, and functional levels. Data are being accumulated on the effect of sex hormones on TRP channels such as TRPV1 that make pivotal contributions to nociceptor excitability and sensitization in migraine and other chronic pain syndromes. These data suggest that modulation of TRP channels' expression and/or activity by gonadal hormones provide novel pathways for drug intervention that may be useful for targeting the sex dimorphism observed in migraine.

## Introduction

Chronic pain is a disease that affects more than 20% of the world's population (Breivik et al., 2006; Nahin, 2015), and is considered a social, medical, and economic burden (Patapoutian et al., 2009). Chronic pain results from complex processing of molecular and cellular signals at different levels of the peripheral (PNS) and central nervous systems (CNS), and also involves the immune system. A plethora of molecules and signaling pathways are involved in the detection, transduction, and propagation of environmental noxious stimuli by nociceptors, a specialized class of sensory neurons. These include members of the superfamily of Transient Receptor Potential (TRP) channels, which act as molecular sensors of harmful chemical and physical stimuli.

The TRP channels are non-selective cation channels expressed mainly in the plasma membrane of different cell types, as well as in the membrane of some intracellular organelles (Venkatachalam and Montell, 2007). They are implicated in a variety of sensorial functions, expanding from vision and taste to nociception (Clapham, 2003; Venkatachalam and Montell, 2007; Julius, 2013; Jardin et al., 2017). Structurally, the TRP channels are tetrameric integral membrane proteins whose monomeric subunits display a domain structure characterized by six transmembrane segments (S1-S6), C- and N-cytosolic domains, and an aqueous pore region structured by S5 and S6 segments and their connecting loop. Both C- and N-termini exhibit particular functional domains depending on the subfamily (Ferrer-Montiel et al., 2004; Latorre et al., 2009; Cao et al., 2013; Liao et al., 2013). Currently, the TRP family is divided into six subfamilies according to sequence homology, namely canonical (TRPC), vanilloid (TRPV), ankyrin (TRPA), melastatin (TRPM), polycystic (TRPP), and mucolipin (TRPML) (Venkatachalam and Montell, 2007; Flockerzi and Nilius, 2014). In mammals, a total of 28 members have been identified, of which 4 members of TRPV (TRPV1-4), 2 members of TRPM (TRPM8 and TRPM3), and the only member of the TRPA subfamily (TRPA1) are known as thermoTRP channels, as they are environmental temperature sensors. Notably, thermoTRPs have been related to the pathophysiology of pain (Mickle et al., 2015, 2016).

Activation of thermoTRP channels in nociceptors provokes a Na^+^ and Ca^2+^ influx resulting in membrane depolarization, neuronal exocytosis, and action potential firing, as well as activation of intracellular second messenger cascades that may lead to neuronal adaptation, i.e., desensitization and/or potentiation (Ramsey et al., 2006; Ciardo and Ferrer-Montiel, 2017). All thermoTRPs are polymodal channels, being gated by a variety of chemical and physical stimuli, such as temperature, pH, osmolarity, endogenous compounds, inflammatory molecules, and natural products (Nilius and Szallasi, 2014; Dai, 2016; Moran and Szallasi, 2017). Natural compounds, such as capsaicin, menthol, and cinnamaldehyde, have been widely used to explore and understand the role of these channels in nociception and pain (Julius and Basbaum, 2001; Julius, 2013).

The ThermoTRP channels are functionally modulated by lipids (Ciardo and Ferrer-Montiel, 2017). Cholesterol has been described to be a modulator of TRPV and TRPM channels, involved in potentiating or reducing their activity (Taberner et al., 2015; Morales-Lazaro and Rosenbaum, 2017). One family of molecules derived from cholesterol metabolism is steroid hormones (Hu et al., 2010). Progestogens, estrogens, and androgens are powerful molecules that regulate a wide variety of cellular functions, and the link between them and pain vulnerability continues to grow. Several reports point to a transcriptional regulation of thermoTRPs expression by sex hormones (Jung et al., 2009; Kumar and Singru, 2017). Notably, recent studies in mice suggested a direct activation and/or modulation of thermoTRP channels by steroids (Asuthkar et al., 2015a,b; Ortiz-Renteria et al., 2018). Thus far, the link between sex hormones and pain has been mainly centered around how these hormones affect the structures in the CNS related to stress, anxiety, and pain (Aloisi and Bonifazi, 2006). It is well-established that estrogens regulate and modulate the opioid system contributing to analgesia differences in males and females at both experimental and clinical levels (Craft, 2003; Fillingim and Gear, 2004; Loyd and Murphy, 2014). Furthermore, the sex hormones appear to disturb the interrelation between the immune system and opioid receptors (Doyle and Murphy, 2017). Cumulative evidence implies a modulation of the somatosensory system function by sex hormones that may underlie the human gender prevalence in some types of chronic pain (Mapplebeck et al., 2017; Sorge and Totsch, 2017).

As suggested by Kumar et al. (2015), the cross-talk between steroid hormones and thermoTRPs may have important implications for clinical context of human diseases. A relevant case in point is migraine, which is the seventh most prevalent medical disease and the second most disabling neurological condition in the world (GBD 2015 Disease Injury Incidence Prevalence Collaborators, 2016; GBD 2015 Neurological Disorders Collaborator Group, 2017). This episodic primary headache is often characterized by intense, unilateral, throbbing, and pulsatile headache attacks, lasting for 4–72 h, and is frequently accompanied by nausea, vomiting, photophobia, and/or phonophobia. It is commonly divided into two main groups—with and without aura, that correspond to transient focal neurological symptoms that usually precede or sometimes accompany a headache (Headache Classification Committee of the International Headache Society, 2013). Migraine exhibits a clear sex difference in prevalence, indicating a predominant role of sex hormones as triggers *per se* or modulators of headache attacks through regulation of thermoTRP channels. Although both have separately been proposed as therapeutic targets for migraine intervention, the interrelation of sex hormones and thermoTRPs in the etiology of the disease has not been addressed in depth.

Here, we review the role of sex hormones in the activation, modulation, and regulation of the main thermoTRP channels involved in the pathophysiology of migraine. Nonetheless, we should mention that sex differences in migraine, and other chronic pain syndromes, will also be influenced by gonadal-independent X-linked gene expression that contributes to inborn sex differences in organs, tissues, and cells (immune, endothelial, and neurons), as well as by other factors (i.e., psychological and social) (review in Mogil, 2012; Bartley and Fillingim, 2013). The available information on the influence of these gonadal-independent factors on the pathophysiology of migraine, especially on the expression and activity of TRP channels, is very scarce, thus preventing us from properly addressing it in this review. Accordingly, we focus on the information regarding the direct interaction and modulation of thermoTRP channels by sex hormones, which may, at least in part, underlie the greater prevalence of the disease in women. We suggest that thermoTRPs may represent potential therapeutic targets for migraine intervention and other pain syndromes that exhibit sex dimorphism.

## Influence of sex hormones in migraine

Cumulative evidence indicates that migraine is a chronic pain disease linked to sex hormones. Firstly, ~15% of the population suffer from this, including children; however, the prevalence in women is up to three times higher than in men. Although a peak of incidence appears in individuals in the age range of 25–55 years in both genders, this remains higher in women (Stewart et al., 1992; Lipton et al., 2001, 2007; Mathers et al., 2008; Vetvik and MacGregor, 2017). Secondly, the migraine prevalence changes across the age range. In 2003, a National Health Interview Survey, in which more than 40,000 US-citizens (70% adults and 30% children) were interviewed, showed that boys and girls shared a similar 1-year prevalence until puberty, thereafter it increased in both genders, being two or three times greater in women (Victor et al., 2010). This study also found that the largest difference in migraine prevalence occurred at the age of 30.2 years, declining from the age of 42 years (Victor et al., 2010). In women, the prevalence sharply decreased at menopause (Vetvik and MacGregor, 2017). The sex difference in the disease incidence between 15 and 50 years is probably related to the higher level of sex hormones during this age range. Most studies showed a protective role of testosterone and progesterone against migraine crisis, while the data for estrogens were more controversial. There are studies reporting that low levels of estrogens may be related to an increase in the number of migraine attacks, whereas others suggest that the application of estrogens promotes migraine episodes (see below).

In addition to the higher prevalence of migraine in females, it has also been reported that women experience more frequent, longer-lasting, and more intense attacks than men (Celentano et al., 1990; Boardman et al., 2003). The constant finding was that women, in comparison to men, have longer-lasting migraine attacks (Kallela et al., 1999; Steiner et al., 2003; Wober-Bingol et al., 2004; Kelman, 2006; Murtaza et al., 2009; Franconi et al., 2014; Bolay et al., 2015), as well as longer photophobia, phonophobia, nausea, vomiting, and cutaneous allodynia (Steiner et al., 2003; Murtaza et al., 2009; Bolay et al., 2015). One study, which analyzed 2,082 migraine adult patients (1,804 women and 278 men), reported that the headache intensity in women changed in an age-dependent manner and the duration and intensity of each attack achieved a peak above the age of 30 years. None of these variations were detected in men (Bolay et al., 2015). Therefore, these changes in the frequency and/or intensity seemed related to changes in women's reproductive status (puberty, pregnancy, or menopause) (Gupta et al., 2007), which were associated with fluctuating levels of estrogen and progesterone in the menstrual cycle. In support of this tenet, it has been shown that there is a significantly increased risk in women to suffer a migraine episode between 2 days before and 3 days after menstruation, which could be related to the lowest concentration of estrogen and progesterone (reviewed in Gupta et al., 2007; Macgregor, 2014; Vetvik and MacGregor, 2017). Furthermore, the headache classification set out by the Committee of the International Headache Society (2013) indicated that migraine without aura was often related to the menstrual cycle, thus categorizing it as pure menstrual migraine if the attack occurred only during the cycle, and menstrual-related migraine if there were additional episodes with or without aura during the menstrual cycle. Although the first phenomenon is not common (Gupta et al., 2007), menstrual-related migraine has been reported in more than 50% of women with migraine (Martin, 2004). Akin to menstruation, treatments involving the intake of hormonal contraceptives have been related to a higher frequency of migraine episodes (MacGregor, 2013).

Perimenstrual estrogen withdrawal seems to be a trigger for migraine without aura. Based on this finding, perimenstrual estrogen supplements (estradiol patches) to 22 migraineur women significantly reduced the number of menstrual-related migraine attacks and also the intensity of the attacks during the months of treatment, as compared with placebo (Dennerstein et al., 1988). Similarly, in another pilot clinical trial with 20 migraineur women, estradiol gel reduced the duration and intensity of the migraine attacks when compared to a placebo gel (de Lignieres et al., 1986). Interestingly, an inverse association between migraine and estrogen levels in urine was found (MacGregor et al., 2006a). However, this study did not establish a threshold for estrogen withdrawal to trigger a migraine attack. Another study with 21 migraineur women reported that transdermal estradiol patches induced a slightly preventive effect for migraine crisis in women with induced menopause, thereby concluding that a low amount of estradiol in serum is sufficient to evoke migraine (Martin et al., 2003). During pregnancy, when estrogen and progesterone are 10 times higher than in non-pregnant states, an improvement in the disease was reported, especially in women suffering from menstrual-related migraine, although in some cases migraine with aura worsened in the first month of pregnancy (Macgregor, 2014).

The prevalence of migraine during perimenopause (period of 2–8 years prior to menopause and 1 year after the end of menses) appears to be higher among women who had suffered from menstrual-related migraine (Mattsson, 2003; Wang et al., 2003). In a clinic-epidemiologic report, which analyzed 556 migraineur women, two-thirds of women suffering from migraine reported an improvement in the disease after spontaneous menopause. However, it worsened in women after surgical menopause (Neri et al., 1993). During this period, the treatment of choice for migraine was transdermal estrogen patches or an estrogen gel, since there was evidence that oral pharmacological treatment could worsen migraine because of a greater systemic hormonal fluctuation (MacGregor et al., 2006b). Overall, there is an increased risk of migraine during the reproductive years, which decreases in the post-menopause phase (Ripa et al., 2015). Nonetheless, to confirm the effect of menstrual hormones on migraine, longitudinal studies are required.

A study by Li et al. (2018) analyzing 119 migraineurs, 42 patients with tension-type headache, and 30 healthy controls tried to relate sex hormones with migraine clinical outcomes in men and menopausal, perimenopausal, and reproductive-aged women. In this study, testosterone appeared to be lower in all migraineur women categories when compared to healthy controls, while progesterone appeared to be lower in both men and postmenopausal women. High estrogen levels in men and reproductive women appear to correlate with the least incapacitating migraine attacks. However, high estrogen levels were positively related to the duration of the migraine episode in post-reproductive women during the luteal phase (Li et al., 2018). With several limitations, similar to previous prospective studies, this study suggested a complex role of sex hormones in the etiology of migraine.

## Pathophysiology of migraine

Several events that occur in the CNS and PNS during a migraine attack, particularly in the trigeminal ganglia (TG), have been described. To mention a few: (i) a neuronal hyperexcitability in cortical regions (Welch, 2005; Aurora and Wilkinson, 2016); (ii) a cortical spreading depression (CSD) likely connected with the aura phase (Iadecola, 2002; Eikermann-Haerter et al., 2009, 2011; Zhang et al., 2010); (iii) the activation and sensitization of trigeminal nociceptors at the peripheral and central levels (Buzzi and Moskowitz, 1992; Goadsby and Edvinsson, 1993; Bolay et al., 2002); (iv) a cranial vasodilatation and meningeal inflammation (Moskowitz and Macfarlane, 1993; Williamson and Hargreaves, 2001; Levy, 2009).

Some of these events have been reported as a consequence of others, without clarifying which is the main cause or the trigger of a migraine episode. Meningeal blood vessels are located in the dura mater of the meninges in the CNS. The role of these vessels and their vasodilation in migraine has been widely studied (Humphrey and Goadsby, 1994; Goadsby et al., 2002; Parsons and Strijbos, 2003). Various studies in animals indicate that meningeal inflammation is the driving event of nociceptor sensitization (Williamson and Hargreaves, 2001; Levy, 2009, 2012). Accordingly, it was thought that vasodilation of these meningeal blood vessels was responsible for migraine triggers and therefore, some vasoconstrictors were developed to treat acute migraine (Villalon et al., 2003). However, other researches suggested that vasodilation of meningeal blood vessels was the consequence of trigeminal system activation rather than the major trigger. Thus, it was reported that the trigeminal sensitization caused cranial vasodilation in both humans and cats as a result of the production of nitric oxide (NO) that promoted the release of neuropeptides, such as substance P (SP) and α-calcitonin gene-related peptide (α-CGRP) (Goadsby and Edvinsson, 1993; Goadsby et al., 2009). The secretion of these vasoactive neuropeptides underlies the vasodilation characteristic of a migraine attack (Waeber and Moskowitz, 2005). In 2008, Schoonman and colleagues published a paper in which they induced migraine in migraineurs and healthy individuals by injecting the vasodilator nitroglycerin, a NO donor. Contrary to the widespread belief, they observed that only the control subjects experienced vasodilation of the meningeal vessels (Schoonman et al., 2008). Thus, the vasodilation of meningeal vessels seemed not to be the main cause of migraine pathophysiology, although it pivotally contributes to the symptomatology of the disorder. On the other hand, many studies have shown that CSD promotes trigeminal sensitization (Bolay et al., 2002; Zhang et al., 2010). Hence, the precise molecular and cellular mechanisms underlying migraine attacks remain largely elusive (Goadsby et al., 2009).

The triggering event in migraine is still under intense debate (Burstein and Jakubowski, 2005; Levy et al., 2009; Messlinger, 2009; Olesen et al., 2009; Charles, 2010; Levy, 2010, 2012; Bolay, 2012). Several groups postulated that the CNS is the starting point of the migraine attack, but other groups suggested that migraine is initiated by the activation of the trigeminal system. One data point that supports a peripheral triggering event is that during a migraine attack, elevated levels of inflammatory mediators, such as nerve growth factor (NGF), bradykinin, prostaglandins, and eicosanoids, are observed (Goadsby and Edvinsson, 1993; Sarchielli et al., 2006). This “inflammatory soup” has been largely postulated to be a key factor that sensitizes the TRPV1 and TRPA1 channels. Sensitization of these thermoTRP channels promotes the release of αCGRP in trigeminal terminals that in turn induces cranial vasodilatation (Meents et al., 2010; Benemei et al., 2013, 2014). Similarly, the application of capsaicin in the trigeminal system has been used as an experimental model of migraine (Gazerani et al., 2005). However, in a different study, capsaicin was shown as a relief for migraine attacks by depleting vasoactive neuropeptides (Benemei and Geppetti, 2013). Besides capsaicin, other TRP channel agonists have been described as migraine triggers (Kunkler et al., 2011), thereby placing TRPV1, TRPA1, and TRPM8 in the therapeutic spotlight for the development of migraine treatments (Nassini et al., 2010; Oxford and Hurley, 2013; Dussor et al., 2014). Interestingly, some chemical agents such as cigarette smoke, ammonia, formaldehyde, and chlorine can induce migraine attacks—all these compounds are TRPA1 agonists (Benemei et al., 2012). The headache produced by these environmental agents has been shown to be mediated by the secretion of αCGRP that increases cerebral blood flow (Kunkler et al., 2011). Additionally, low levels of magnesium in early embryonic development, which is able to permeate TRPM6 and TRPM7 channels, have also been reported as triggering migraine episodes (Komiya and Runnels, 2015).

In several meta-analysis and genome-wide association studies (GWAS) the TRPM8 locus has been related to susceptibility to migraine (Chasman et al., 2011; Esserlind et al., 2016; Gormley et al., 2016; Key et al., 2018). Also, genetic variations in single nucleotide polymorphisms (SNPs) in TRPV1 and TRPV3 loci in a Spanish cohort were linked to a genetic predisposition to migraine (Carreño et al., 2012) (review in Zorina-Lichtenwalter et al., 2016). Thus, these studies implicate thermoTRPs as pivotal contributors to migraine, and suggest that they may be interesting targets for the treatment of migraine (Dussor et al., 2014; Tso and Goadsby, 2014).

## TRPV1 and estrogens

TRPV1 channels, along with other thermoTRPs, are localized predominatly in peripheral and central nerve terminals of dorsal root ganglia (DRGs) and TGs (Caterina et al., 1997; Ichikawa and Sugimoto, 2001; Peier et al., 2002; Story et al., 2003; Bae et al., 2004; Okazawa et al., 2004; Roberts et al., 2004; Bautista et al., 2005; Kobayashi et al., 2005; Shimizu et al., 2007; Huang et al., 2012). Notably, TRPV1 is highly co-expressed with αCGRP (Ichikawa and Sugimoto, 2001; Bae et al., 2004), as well as with Substance P (SP) and P2X3 purinergic receptors. TRPV1 colocalizes with TRPA1 as well (Story et al., 2003; Bautista et al., 2005), whereas TRPM8 is expressed in a different subpopulation of nociceptors (Peier et al., 2002; Story et al., 2003; Okazawa et al., 2004; Ren et al., 2018).

TRPV1 is also present in the brain, particularly in the hypothalamus, thalamus, amygdala, periaqueductal gray, insula, and a number of other regions in the brain of humans and rodents (Mezey et al., 2000; Roberts et al., 2004; Steenland et al., 2006; Cavanaugh et al., 2011). Interestingly, brain regions expressing TRPV1 are targets of sex hormones. Imaging studies in 44 adult migraineurs (22 women and 22 men) showed that migraineur women exhibited a thicker posterior insula and precuneus cortices compared to male migraineurs and the healthy controls of both sexes (Maleki et al., 2012). The authors suggested that these morphological alterations might underlie the different responses of both genders to migraine attacks, as well as the effect of sexual hormones and the differential impact of anti-migraine drugs such as triptans (Maleki et al., 2012).

In addition to the presence of TRPV1 channels in the brain regions that are influenced by sex hormones, some studies have demonstrated the expression of estrogen receptors (ERα, ERβ y GPR30) in sensory neurons (Papka et al., 1997; Papka and Storey-Workley, 2002; Takanami et al., 2010) where they colocalize with TRPV1 channels (Bennett et al., 2003). This co-expression in nociceptors has led to the hypothesis of a modulation of pain transduction by sexual hormones (Bennett et al., 2003; Chaban, 2013). In support of this hypothesis, there is experimental and clinical evidence relating the function of estrogens with TRPV1 activity. For instance, it has been reported that women experience more pain-related sensations on exposure to TRPV1 agonists than men (Jensen and Petersen, 2006; Gazerani et al., 2007).

Some studies analyzed the effects of steroid hormones on capsaicin-evoked currents in rodent nociceptors. Although a differentiation between sexes was not established, these studies detected that 17β-estradiol, the dominant estrogen during a woman's reproductive phase, is an important enhancer of capsaicin responses evoked *in vitro* in primary cultures of rodent nociceptors (Chen et al., 2004). This observation was also seen *in vivo*, where the threshold of the nociceptive responses to capsaicin injection was significantly reduced in rodent females (Lu et al., 2009). Although both sexes showed sensitivity to capsaicin, males required a four-fold higher dose of capsaicin than females for a similar response (Lu et al., 2009). It should be noted that the differences in capsaicin sensitivity observed between sexes may be caused by differential estrogen levels and influenced by the distinct percentage of estrogen receptors in male and female nociceptors (Takanami et al., 2010).

When estrogen levels were monitored, either as a function of the estral cycle or its replacement after ovariectomy in rodents, it was observed that under low levels of 17β-estradiol, capsaicin produced a mild nocifensive response (Lu et al., 2009; Yamagata et al., 2016). In marked contrast, high levels of 17β-estradiol (proestrus) resulted in a lower mechano- and thermo-nociceptive threshold, thereby promoting mechanical and thermal sensitization (Payrits et al., 2017). This nociceptive sensitization was not observed in TRPV1 knock-out mice, implying a link between estrogen nociceptor sensitization and TRPV1 *in vivo* (Payrits et al., 2017).

At the cellular level, it has been proposed that nociceptor sensitization induced by estrogens could be provoked by an alteration of nociceptor excitability. Flake et al. (2005) demonstrated that estrogens reduced the threshold of action potential firing and increased their spontaneous activity. They speculated that these changes in nociceptor excitability may be associated with membrane depolarization provoked by estrogen-induced activation of thermoTRP channels, particularly TRPV1. In this study, however, the authors did not find a significant difference in the proportion of nociceptors that responded to capsaicin between ovariectomized females treated with vehicle and those treated with estrogens after ovariectomy (Flake et al., 2005). In contrast, Yazgan and Naziroglu (2017) showed that ovariectomy led to higher capsaicin-induced current densities. Replacement therapy with 17β-estradiol reduced capsaicin responses to a level similar to that of mock-operated animals (Yazgan and Naziroglu, 2017).

Curiously, some studies show that TRPV1 sensitization by 17β-estradiol was stereospecific. However, the 17β-estradiol induced TRPV1 potentiation, the stereoisomer, 17α-estradiol, did not affect the activity of the channel in sensory neurons (Chen et al., 2004; Lu et al., 2009). Similarly, 17α-estradiol did not affect the vasorelaxation induced by anandamide, an endogenous TRPV1 agonist (Ho, 2013). It should be noted, however, that this observation is still under debate as this stereospecificity was not observed in another study (Xu et al., 2008).

The molecular mechanisms involved in 17β-estradiol-induced sensitization of TRPV1 appeared to include both a genomic regulation of channel expression and a functional modulation. Regarding the long-term effect of estrogens in females, several studies showed a transcriptional-induced expression of TRPV1 by 17β-estradiol in nociceptors (Yamagata et al., 2016; Kumar et al., 2017; Payrits et al., 2017). Even sensory neurons derived from female mouse stem cells showed that the application of 17β-estradiol increased the expression of TRPV1 mRNA (Greaves et al., 2014). Similarly, Cho and Chaban (2012) published another study that showed the relationship between estrogens and TRPV1 expression. Notably, they reported a reduction of TRPV1 expression in ERα and ERβ null lines. Estradiol-induced TRPV1 expression has been observed not only in neuronal samples (Wu et al., 2010; Kumar and Singru, 2017) but also in non-neuronal tissues such as peritoneum (Greaves et al., 2014), endometrium (Pohoczky et al., 2016), and synoviocytes (Wu et al., 2015). Although still limited, some genomic studies imply the presence of a putative functional estrogen response element in the TRPV1 promoter (Greaves et al., 2014; Kumar and Singru, 2017).

**Figure 1:**
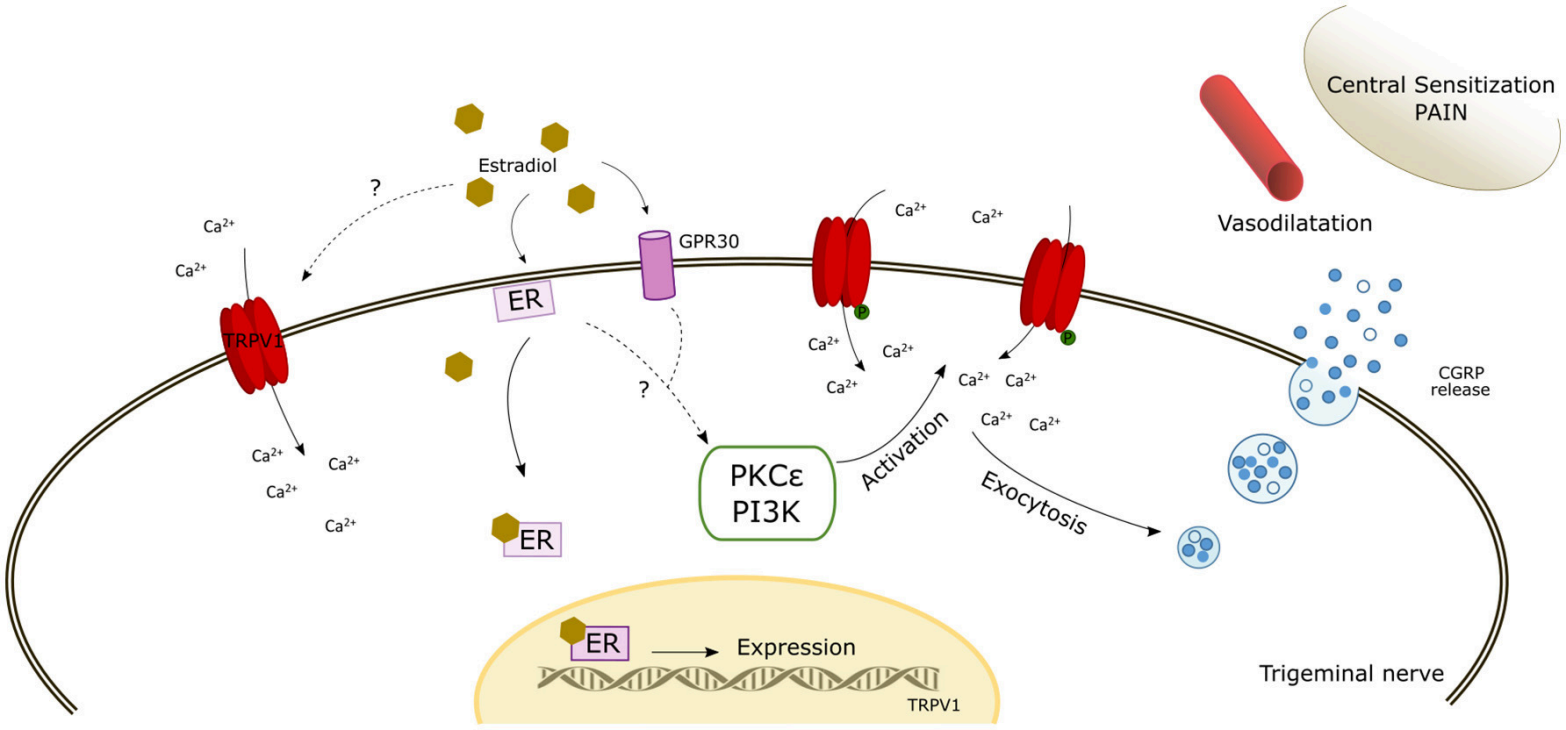
Schematic diagram of the potential mechanisms of action of estrogen modulating TRPV1. The scheme reflects a possible direct activation by estradiol. In addition, the interaction of estradiol with estrogen receptors (ER and GPR30) may induce the expression of TRPV1 and likely activate intracellular signaling pathways that phosphorylate TRPV1 inducing its membrane expression and its activation/sensitization. The increase of intracellular calcium enhances αCGRP release which induces cranial vasodilatation, trigeminal sensitization, and pain. ER, estrogen receptor; PKCε, Protein Kinase C epsilon; PI3K, Phosphatidylinositol-4,5-bisphosphate 3-kinase.

In addition to the genomic regulation, there are several studies that have provided evidence for the modulation and/or a direct effect of 17β-estradiol on TRPV1 based on the rapid responses that can be induced by estrogens. An *in vivo* study in rodents reported a fast onset of 17β-estradiol on capsaicin sensitization when instilled locally in the ipsilateral paw, without an effect on the contralateral paw (Lu et al., 2009). Furthermore, pre-incubation with PKC or PKA inhibitors did not enhance capsaicin responses, thereby suggesting a direct effect of 17β-estradiol on TRPV1 activity (Lu et al., 2009). In support of this, other studies have suggested that the effect of estrogens on TRPV1 activity was mediated by PKCε phosphorylation of Ser-800, which enhances channel activity (Hucho et al., 2006; Kuhn et al., 2008; Goswami et al., 2011). Through a TRPV1-dependent mechanism, but independent of its ion channel activity, PKCε may promote cytoskeletal destabilization that in turn may produce mechanical nociceptor sensitization (Goswami et al., 2011). In contrast, Payrits et al. (2017) suggested that 17β-estradiol-induced TRPV1 sensitization in nociceptors may be indirectly produced through the TrkA pathway as the application of inhibitors of this pathway abolished TRPV1 sensitization by estrogen (Figure 1).

However, to add more confusion to the field, not all studies have shown that 17β-estradiol enhances the expression and sensitization of TRPV1 channel in nociceptors. Indeed, there are studies that imply a preventive or analgesic effect of estrogens. For instance, Yazgan and Naziroglu (2017) observed that deprivation of 17β-estradiol was related to an excessive production of mitochondrial oxygen free radicals and a high Ca^2+^ influx through TRPV1, TRPA1, and TRPM2 channels, which appears to be one of the main causes of neurodegenerative disease in postmenopausal women. This study also showed that TRPV1 levels decrease as a result of 17β-estradiol administration, and ovariectomy produced capsaicin sensitization in line with a previous study (Sanoja and Cervero, 2005). Another study reported that a long-term incubation with 17β-estradiol reduced the activation of TRPV1 (Xu et al., 2008). Similarly, Thompson et al. (2008) pointed out that the anti-nociceptive action of 17β-estradiol depended on the concentration used, as high doses of 17β-estradiol benzoate produced a low nociceptive response in females. Clearly, additional studies are needed to further unveil estrogen-mediated modulation of thermoTRPs in sensory neurons and other cell types involved in migraine. Furthermore, attention has to be paid to the experimental conditions used in *in vitro* studies as these may have a notable influence on the final modulatory effect of gonadal hormones. For instance, nociceptor subpopulations should be considered, as they may exhibit differential sensitivity to these hormones.

## TRPV1 and progesterone

Progesterone is the other steroid hormone that, together with estradiol, regulates the estral cycle in rodents and the menstrual cycle in humans. There are a plethora of studies linking progesterone to anti-nociception in neuropathic pain models (Coronel et al., 2011, 2016; Verdi et al., 2013; Jarahi et al., 2014; Liu et al., 2014). In this context, the few studies that investigated the putative effects of progesterone in the expression and functional modulation of thermoTRPs showed mainly an inhibitory role of this hormone. For example, it was observed that progesterone, acting through its receptor, reduced the expression of TRPV4 in sensory neurons (Jung et al., 2009). Recently, Ortiz-Renteria et al. (2018) reported a new molecular mechanism through which progesterone and the Sig-1R chaperon promoted TRPV1 down-regulation in nociceptors. The interaction of progesterone with the Sig-1R chaperon inhibited its binding to the TRPV1 promoter, resulting in a transcriptional inhibition of TRPV1 expression and consequently a lower nociceptive response to capsaicin. This *in vivo* observation is in disagreement with Chen et al. (2004) who observed that progesterone did not modify capsaicin-induced currents in DRGs cultures. Similarly, Lu et al. (2009) found that the therapeutic replacement of progesterone in ovariectomized rats did not change the nocifensive response to capsaicin application.

Akin to estrogens, it is plausible that progesterone nociceptive effects are concentration-dependent, being stronger at high concentrations. This may account for the lack of a clear anti-nociceptive effect of the hormone during the estral cycle, where it is present at low concentration. On the other hand, progesterone seemed to mediate anti-nociceptive responses during pregnancy (Ortiz-Renteria et al., 2018). Furthermore, progesterone is a direct agonist of TRPM3 channels (Majeed et al., 2012; Miehe et al., 2012; Kumar et al., 2015) and it appears to regulate TRPC3 channels (Majeed et al., 2011). As with estrogens, more studies are needed to fully understand the potential role of progesterone modulation of nociceptor excitability in contributing to migraine episodes.

## TRPV1 and other sex-related hormones

Prolactin is a peptide hormone secreted by the anterior pituitary gland that plays a role in osmoregulation, metabolism, the immune system, and the stimulation of lactogenesis and galactopoiesis. Prolactin is also involved in inflammatory responses (Costanza et al., 2015; Pereira Suarez et al., 2015). In TG neurons, a prolactin receptor is highly expressed in TRPV1 nociceptors, where it potentiates capsaicin-evoked currents, calcium influx, and αCGRP release (Patil et al., 2014). Diogenes et al. (2006) reported that 17β-Estradiol produced a notable increase of prolactin in TG overlapping with TRPV1-expressing sensory neurons. Notably, prolactin enhanced the capsaicin-evoked responses in TG nociceptors, both *in vitro* and *in vivo*, in a 17β-Estradiol-dependent manner. Furthermore, prolactin significantly augmented the capsaicin-induced nociceptive responses in female rats at proestrus and in ovariectomized females after estradiol treatment (Diogenes et al., 2006). In a subsequent study, the same group demonstrated that the short prolactin receptor signaling pathway mediated the activation of TRPV1 via PKC and PI3K, thereby affecting the action potential threshold and excitability of nociceptors (Belugin et al., 2013; Patil et al., 2013a,b, 2014).

In addition to prolactin, there is increasing interest in oxytocin because it may become an analgesic target for various chronic pain pathologies due to its role in pain modulation (Gonzalez-Hernandez et al., 2014; Rash et al., 2014; Tracy et al., 2015; Valstad et al., 2016). The nociceptive modulation exerted by this neuropeptide is mediated through two main pathways. Firstly, the endogenous opioid system plays an important indirect role in the modulation of pain by oxytocin. Specifically, the activation of opioid receptors appears to drive oxytocin central analgesic effects (Rash et al., 2014). Secondly, the activation of GABAergic inhibitory interneurons directly inhibits nociceptive C and Aδ fibers at the spinal cord (Gonzalez-Hernandez et al., 2014; Rash et al., 2014). The relative importance of this system remains elusive because it is still unknown whether there are oxytocin receptors in nociceptors. In addition, the peripheral contribution of vasopressin 1a receptors might also explain the oxytocin nociception regulation. Clearly, information about the receptors involved in the antinociceptive effects of oxytocin, at the peripheral nociceptors endings and supraspinal levels, remains a key research area in pain science (Gonzalez-Hernandez et al., 2014), which requires urgent investigation.

There are several attempts to apply intranasal oxytocin as an analgesic therapy, and even as a migraine treatment (Wang et al., 2013; Tracy et al., 2015). However, the intranasal application of oxytocin is often not effective. For instance, some women reported an increase in the perceived intensity of noxious heat stimuli after oxytocin inhalation (Tracy et al., 2017). In this context, a recent report describing, in both sexes, the direct agonist action of oxytocin on TRPV1 is a significant step toward disentangling the mechanism of action of oxytocin on nociceptors (Nersesyan et al., 2017). These results provide an explanation for the hot pain intensity reported by women patients under oxytocin therapy. Furthermore, oxytocin anti-nociceptive activity may be due to TRPV1 desensitization in nociceptors, similar to that induced by capsaicin and resiniferatoxin. Interestingly, the different physicochemical characteristics of oxytocin and capsaicin suggest a distinct receptor binding site for the hormone. However, it appears that oxytocin may bind to an outer transmembrane site located in the interfacial region between two adjacent subunits (Nersesyan et al., 2017), distant from the capsaicin binding site (Cao et al., 2013; Darre and Domene, 2015; Yang and Zheng, 2017). The discovery of this novel binding site in the TRPV1 channel opens new avenues for the design of novel receptor antagonists for the treatment of migraine.

## TRPV1, TRPM8, and testosterone

The lower prevalence of chronic migraine in men is also characterized by a lower intensity and/or shorter duration of pain symptoms as compared to women (Bartley et al., 2015), thereby suggesting a role of testosterone. There are clinical studies showing that testosterone replacement therapy reduces pain outcomes and improves the quality of life of patients with hypogonadism (Aloisi et al., 2011), thereby supporting the hypothesis that testosterone may have an anti-nociceptive role. However, some studies suggest that testosterone is necessary for a small nociceptive response (Thompson et al., 2008; Glaser et al., 2012; Schertzinger et al., 2017). According to these studies, low levels of testosterone are related to high discomfort, anxiety, and pain in response to noxious hot stimuli (Choi et al., 2017). In another study, Fanton et al. (2017) concluded that the protective effect of testosterone is due to the activation of androgen receptors by the hormone instead of an androgenic action of a testosterone derivative (i.e., dihydrotestosterone) during CNS development. These observations are in agreement with the testosterone effect observed in nociceptors. For example, the application of androgenic hormones to nociceptors showed an inhibitory effect on capsaicin-induced currents (Chen et al., 2004). In addition, a recent study observed the significant differences between male and female mice in the expression of TRPV1 channels in TG, after an inflammatory insult, and provided data suggesting that testosterone may be an important contributor to the sensitization of TRPV1 in chronic inflammatory pain (Bai et al., 2018). This study observed a decrease of TRPV1 expression with testosterone replacement after gonadectomy. Although more studies are needed, this report further supports that sex hormones play a central role in modulation of the activity of TRPV1 channels.

Apart from TRPV1, it is worth mentioning that testosterone is also a regulator of TRPM8, a thermoTRP channel involved in cold nociception. Zhang and Barritt (2004) found that the promoter of TRPM8 contains a putative androgen-response element motif. Notably, this channel is highly expressed in the prostate and appears overexpressed in prostate cancer by a testosterone-mediated mechanism. The dependence of TRPM8 expression on androgens was demonstrated, which showed that the replacement of testosterone after gonadectomy induced the recovery of TRPM8 expression (Yang et al., 2012). Testosterone and TRPM8 are not just linked by genomic regulation, as testosterone is a potent TRPM8 agonist (Asuthkar et al., 2015a,b,c). These studies report the first evidence describing an endogenous modulator of this thermoTRP channel, although additional studies are needed to unveil the contribution of testosterone to cold allodynia and other nociceptive conditions promoted by TRPM8 activity. It is worth noting that TRPM8 has been recently proposed as an interesting therapeutic target for migraine due to the analgesic ability of some of its agonists (Dussor and Cao, 2016).

## Conclusions

There is no doubt about the existence of a sex difference prevalence in chronic pain conditions such as migraine, where the prevalence in women is two or three times greater than in men. Although the specific molecular and cellular mechanisms underlying this sex dimorphism are still under intense investigation, a pivotal role of sex hormones regulating the somatosensory system appears clear. It was believed that sex hormones mainly acted to regulate the immune system, but evidence is building up on a direct role modulating nociceptor signaling. This modulation appears mediated by the action of these hormones on thermoTRP channels, such as TRPV1, TRPA1, TRPM3, TRPV4, and TRPM8, and probably others that are still to be investigated. Sex hormones can regulate the expression of these channels, acting at a transcriptional level and/or their channel activity and/or through activation of intracellular signaling pathways that sensitize their activity. Understanding the role of sex hormones modulating the somatosensory system, and unraveling their impact on the long-term nociceptor excitability that underlies chronic pain, will pave the way to the design and development of novel and more efficient therapies that consider sex differences in pain perception. Nonetheless, it should be taken into consideration that sex hormones may not be the only players in determining sexual dimorphism in migraine pain, as this is a very complex phenomenon involving both gonadal-dependent and independent mechanisms that most likely complement each other in defining sex differences in migraine and chronic pain disorders.

## Author contributions

MA-M and SG-R have written parts of the review. AF-M conceived the project and has reviewed and edited the manuscript.

### Conflict of interest statement

The authors declare that the research was conducted in the absence of any commercial or financial relationships that could be construed as a potential conflict of interest.
